# Virtual reality support during systemic cancer therapy to improve anxiety/depressive symptoms and reduce toxicity in patients with gastrointestinal cancers—OncoVR

**DOI:** 10.1016/j.esmogo.2025.100135

**Published:** 2025-02-03

**Authors:** S. Kasper, S. Liszio, K. Schorrmann, M. Gerigk, S. Jovic, O. Basu, K. Kostbade, B. Goraus, A. Elsakka, B. Puladi, J. Kleesiek, M. Schuler, G. Luijten, J. Egger

**Affiliations:** 1 Department of Medical Oncology, West German Cancer, University Hospital Essen, Essen, Germany; 2 National Center for Tumor Diseases (NCT) West, Essen, Germany; 3 The Center for Virtual and Extended Reality in Medicine, University Hospital Essen, Essen, Germany; 4 Institute for Artificial Intelligence in Medicine, University Hospital Essen, Essen, Germany; 5 Institute for Patient Experience, University Hospital Essen, Essen, Germany; 6 Institute of Medical Informatics, University Hospital RWTH Aachen, Aachen, Germany

**Keywords:** virtual reality, cancer therapy, supportive care, clinical trial, patient experience

## Abstract

**Background:**

Systemic cancer therapy may trigger anxiety/depressive symptoms and toxicity. Relaxation techniques can help alleviate toxicities but their implementation in clinical practice is challenging. We hypothesize that virtual reality (VR) systems which project a relaxing nature environment may help to reduce psychological stress and toxicities of cancer therapies. This trial aims to evaluate the feasibility of a supportive VR intervention in patients receiving cancer therapies in an outpatient setting.

**Patients and methods:**

OncoVR is a randomized, open-label, cross-over trial to investigate the feasibility and impact of VR support during cancer therapy to improve anxiety, depressive symptoms, and toxicity in patients with gastrointestinal cancers. In total, 54 participants will be assigned to receive systemic therapy with VR support, followed by a subsequent course without VR support (arm A). Patients in arm B will first receive therapy without VR support, followed by a subsequent course with VR support. Primary endpoints are the feasibility of VR support (80% of the patients can tolerate its use for a minimum duration of 20 min), and changes in anxiety/depressive symptoms using the Hospital Anxiety and Depression Scale (HADS-D) and Positive and Negative Affect Schedule (PANAS) questionnaires. Secondary endpoints include the incidence and severity of therapy-associated toxicities per National Cancer Institute Common Terminology Criteria for Adverse Events (NCI-CTCAE) and Patient-Reported Outcomes version of the CTCAE (PRO-CTCAE) grading, and patient experience using the Player Experience Inventory (PXI) questionnaire.

## Highlights

• OncoVR addresses the unmet need for psychological support during cancer therapy.
• OncoVR investigates the feasibility of VR in cancer patients.
• OncoVR may help optimize supportive care protocols in oncology.

## Graphical abstract

**Figure:**
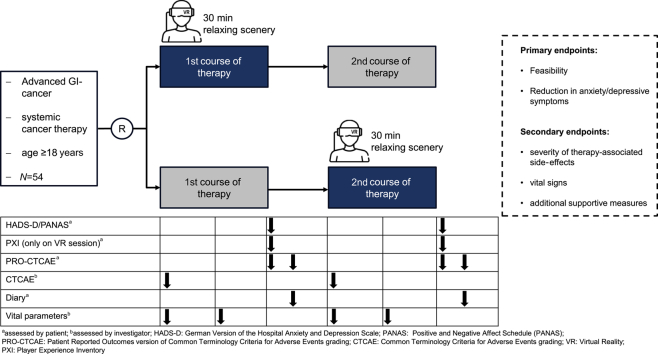

**Study Design Chart Overview**

**Title:** Study Design for VR Support During Systemic Chemotherapy

**Chart Structure:**

**Table:** Specifications table

Subject area	Medicine and Dentistry
**More specific subject area**	Psychological support in oncology.
**Name of your trial in progress**	OncoVR—virtual reality support during systemic cancer therapy to improve anxiety/depressive symptoms and reduce toxicity in patients with gastrointestinal cancers.
**Reagents/tools**	Meta Quest 3 VR headsets (Meta Platforms, Inc.) Nature Treks VR (Greener Games Ltd).
**Trial design**	OncoVR is randomized, open-label, cross-over trial assessing the feasibility and the impact of VR on anxiety/depressive symptoms and toxicity during infusional cancer therapy. In total, 54 participants will be assigned to receive one course of systemic therapy with VR support followed by a subsequent course without VR support (arm A). Patients in arm B will first receive systemic therapy without VR support, followed by a subsequent course with VR support. Primary endpoints are the feasibility and symptom reduction, evaluated using validated questionnaires. Secondary endpoints will include the incidence and severity of therapy-associated toxicity and patient experience including emotional, physical, and psychological impressions.
**Trial registration**	DRKS00035719
**Ethics**	The study is conducted with the Declaration of Helsinki. Informed consent will be obtained from all participants before enrolment. The trial was approved by the Ethics Committee of the Medical Faculty of the University Duisburg-Essen (Project No. 23-11671-BO).
**Value of the Trial in Progress**	− Addresses the unmet need for improved psychological support during systemic cancer therapy. − Investigates the effectiveness of VR technology in reducing anxiety and improving quality of life for cancer patients. − Aims to provide insights that may help optimize supportive care protocols in oncology.

## Description of protocol

### Background

The treatment of patients with advanced cancer undergoing systemic cancer therapy is often associated with significant toxicity and psychological distress.^1^ Although relaxation techniques can alleviate these side-effects, their application in overloaded oncology outpatient clinics is challenging.^2^ The objective of this study is to evaluate the feasibility and impact of using virtual reality (VR) during systemic cancer therapy. The deployment of VR technology may help alleviate anxiety and depressive symptoms, potentially improving patients’ overall quality of life. Previous studies have shown that VR can be an effective support tool in managing anxiety and fatigue in cancer patients, highlighting its potential benefits in oncological care.^3^, ^4^, ^5^

## Study design

OncoVR is a randomized, open-label, cross-over trial to investigate the feasibility and the potential of a VR support during systemic cancer therapy to improve anxiety, depressive symptoms, and toxicity in patients with gastrointestinal cancers. In total, 54 participants will be assigned to receive systemic therapy with VR support, followed by a subsequent course without VR support (arm A). Patients in arm B will first receive one course of systemic therapy without VR support, followed by a subsequent course with VR support. Primary endpoints are the feasibility of using VR technology (80% of patients can tolerate its use for a minimum duration of 20 min); and documenting any improvement in anxious/depressive symptoms using the standardized German versions of Hospital Anxiety and Depression Scale (HADS-D) and the Positive and Negative Affect Schedule (PANAS) questionnaire.^6^^,^^7^ Secondary endpoints include the incidence and severity of therapy-associated toxicities, vital signs, patient satisfaction with the VR support, and the use of additional supportive measures. Therapy-associated toxicity will be assessed per National Cancer Institute Common Terminology Criteria for Adverse Events (NCI-CTCAE) grading by the investigators and the Patient-Reported Outcomes version of the CTCAE (PRO-CTCAE®) questionnaire^8^ by the patients (Supplementary Data, available at https://doi.org/10.1016/j.esmogo.2025.100135). Emotional experience including emotional, physical, and psychological impressions and satisfaction with the VR experience of the players are determined using the Player Experience Inventory (PXI) questionnaire (German version), which is answered after the VR session.^9^ Besides, any additional supportive measures utilized by the patients will be recorded. These would include any antiemetic and analgesic supportive medications taken. Subgroup analyses, including the type of systemic anticancer therapy and pre-medications are planned for the primary and secondary endpoints.

## Statistical considerations

This is an exploratory study, as there are currently limited data regarding an effect size due to the use of VR support, preventing it from being designed as a confirmatory study. The primary endpoints relate to the feasibility of the protocol and any reduction in anxiety and depressive symptoms.

The feasibility of using VR support will be evaluated based on whether at least 80% of the patients can tolerate its use for a minimum duration of 20 min. If only ≤50% of the patients can tolerate the VR support for this period, the implementation will be considered not feasible. Considering these parameters for feasibility, the project requires a sample size calculation with a p1 value of 0.8 and p2 value of 0.5 with a significance level (α) of 0.05 and power of 0.80. This results in a calculated sample size of 46. Accounting for a projected drop-out rate of ∼15%, we aim to recruit a total of 54 patients for the study to ensure a robust dataset for analysis.

Regarding the co-primary endpoint: the reduction of anxiety/depressive symptoms will require the following sample size: a minimal effect size of 3 points in the HADS-D self-assessment scale between the two groups will be considered a significant improvement in symptoms.^6^ At a significance level of α = 0.05 and with a power of 80%, a maximum standard deviation of 3 is assumed for the HADS-D score. A conservative estimate would be 3.5, which leads to a necessary sample size of 24 per study arm for the non-parametric Mann–Whitney *U* test.^10^ Thus, with the calculated total sample size of 54 patients, or 27 patients per arm, the co-primary endpoint can also be evaluated.

## Patient population

The study includes patients who are >18 years old and are diagnosed with gastrointestinal tumors undergoing infusional cancer treatment. The patients’ suitability will be assessed based on their mental and physical well-being to ensure they are capable of taking part in the study procedures. The patients should also be consent-capable, and they must provide signed written consent. Those with contraindications against the use of VR support (i.e. history or diagnosis of a disorder causing seizures) or those participating in another interventional clinical trial, however, will be excluded from the study.

## Study trial treatment

After thorough patient counseling, the inclusion and exclusion criteria are reviewed by the investigator. Following this, patients are randomized and assigned to one of two arms. Patients in arm A will receive systemic therapy with VR support, followed by a subsequent course without VR support (typically 14-21 days later). Conversely, patients in arm B will first receive one course of systemic therapy without VR support, followed by a subsequent course with VR support (also typically 14-21 days later). For this study, the Meta Quest 3 has been selected as the VR support of choice. The VR support will be employed for 30 min at the beginning of the chemotherapy infusion, during which patients will experience a relaxing environment (e.g. beach or forest scene). In this study, we used ‘Nature Treks VR’, developed by Greener Games, a virtual nature simulation designed to provide a peaceful, calming environment. Users can explore diverse ecosystems (e.g. a forest, a beach, the savannah) and interact with various elements (i.e. virtual animals) in the environment. For more information on this application, you can visit their official website: https://www.greenergames.net/nature-treks. This immersive experience is expected to provide distraction and improve comfort during treatment.^5^

Before the therapy, patient, tumor, and treatment characteristics will be collected as part of routine care. This information will be helpful to exclude confounders and to carry out explorative subgroup analyses. Vital parameters such as blood pressure and heart rate will be measured at the start of the VR application and again after 10 min during the experience. Changes in heart rate and blood pressure during the VR application, as autonomic symptoms, will be recorded to further assess the relaxation. If the VR application needs to be terminated early, the reasons for this will be documented. After the VR experience, patients will complete the PXI questionnaire to assess their experience with the VR support.^9^ After completion of the infusional cancer therapy, patients will be asked to rate their perceived anxiety and depressive symptoms and their positive and negative emotions using the German versions of the HADS-D and PANAS questionnaire.^6^^,^^7^ Additionally, 2 days after therapy, patients will complete the PRO-CTCAE questionnaire to assess therapy-related toxicity such as nausea, vomiting, pain, and anxiety.^8^ Treatment-related adverse events per National Cancer Institute Common Terminology Criteria for Adverse Events (NCI-CTCAE) will be documented by the treating physician at each visit. Patients will also receive a treatment diary to document any analgesic and antiemetic medications taken at home. In the therapy session without VR support, only the PXI questionnaire will be omitted; all other procedures and questionnaires will be conducted as planned.

## Declaration of generative AI and AI-assisted technologies in the writing process

During the preparation of this work the author(s) used ChatGPT 4.0 in order to improve readability and language. After using this tool/service, the author(s) reviewed and edited the content as needed and take(s) full responsibility for the content of the publication.
