# Supplementary material for: Virtual reality support during systemic cancer therapy to improve anxiety/depressive symptoms and reduce toxicity in patients with gastrointestinal cancers—OncoVR
Source: ESMO Gastrointest Oncol. 2025 Feb 3;7:100135. doi: 10.1016/j.esmogo.2025.100135 (PMC12836744; doi:10.1016/j.esmogo.2025.100135)
Supplement: Supplementary Data [file mmc1.pdf]

Universitätsmedizin Essen
Westdeutsches Tumorzentrum Essen

Name:
Geb. am.
Patientennummer:
aktuelles Datum bei Ausgabe:

Während einer Krebstherapie können Patienten und Patientinnen verschiedene Symptome und Nebenwirkungen erfahren. Bitte beantworten Sie die folgenden Fragen, indem Sie die Antwort auswählen, die Ihre Erfahrung in den letzten sieben Tagen am besten beschreibt ...

**1a.** Während der letzten 7 Tage: Wie STARK waren Ihre SCHWIERIGKEITEN BEIM SCHLUCKEN im SCHLIMMSTEN FALL?				
O Gar nicht	O Ein wenig	O Mäßig	O Ziemlich	O Sehr

**2a.** Während der letzten 7 Tage: Wie STARK waren Ihre GESCHMACKSVERÄNDERUNGEN BEIM ESSEN ODER TRINKEN im SCHLIMMSTEN FALL?				
O Gar nicht	O Ein wenig	O Mäßig	O Ziemlich	O Sehr

**3a.** Während der letzten 7 Tage: Wie STARK war Ihr APPETITMANGEL im SCHLIMMSTEN FALL?				
O Gar nicht	O Ein wenig	O Mäßig	O Ziemlich	O Sehr
**3b.** Während der letzten 7 Tage: Wie sehr hat Ihr APPETITMANGEL Sie in Ihren täglichen Aktivitäten GESTÖRT?				
O Gar nicht	O Ein wenig	O Mäßig	O Ziemlich	O Sehr

**4a.** Während der letzten 7 Tage: Wie HÄUFIG hatten Sie ÜBELKEIT?				
O Nie	O Selten	O Gelegentlich	O Häufig	O Fast immer
**4b.** Während der letzten 7 Tage: Wie STARK war Ihre ÜBELKEIT im SCHLIMMSTEN FALL?				
O Gar nicht	O Ein wenig	O Mäßig	O Ziemlich	O Sehr

**5a.** Während der letzten 7 Tage: Wie HÄUFIG mussten Sie ERBRECHEN?				
O Nie	O Selten	O Gelegentlich	O Häufig	O Fast immer
**5b.** Während der letzten 7 Tage: Wie STARK war Ihr ERBRECHEN im SCHLIMMSTEN FALL?				
O Gar nicht	O Ein wenig	O Mäßig	O Ziemlich	O Sehr

**6a.** Während der letzten 7 Tage: Wie STARK war Ihre VERSTOPFUNG im SCHLIMMSTEN FALL?				
O Gar nicht	O Ein wenig	O Mäßig	O Ziemlich	O Sehr

**7a.** Während der letzten 7 Tage: Wie HÄUFIG hatten Sie DURCHFALL?				
O Nie	O Selten	O Gelegentlich	O Häufig	O Fast immer

**8a.** Während der letzten 7 Tage: Wie HÄUFIG hatten Sie BAUCHSCHMERZEN?				
O Nie	O Selten	O Gelegentlich	O Häufig	O Fast immer
**8b.** Während der letzten 7 Tage: Wie STARK waren Ihre BAUCHSCHMERZEN im SCHLIMMSTEN FALL?				
O Gar nicht	O Ein wenig	O Mäßig	O Ziemlich	O Sehr
**8c.** Während der letzten 7 Tage: Wie sehr haben BAUCHSCHMERZEN Sie in Ihren täglichen Aktivitäten GESTÖRT?				
O Gar nicht	O Ein wenig	O Mäßig	O Ziemlich	O Sehr

**9a.** Während der letzten 7 Tage: Wie STARK war Ihr SCHWINDEL im SCHLIMMSTEN FALL?				
O Gar nicht	O Ein wenig	O Mäßig	O Ziemlich	O Sehr
**9b.** Während der letzten 7 Tage: Wie sehr hat SCHWINDEL Sie in Ihren täglichen Aktivitäten GESTÖRT?				
O Gar nicht	O Ein wenig	O Mäßig	O Ziemlich	O Sehr

**10a.** Während der letzten 7 Tage: Wie STARK VERSCHWOMMEN haben Sie im SCHLIMMSTEN FALL GESEHEN?				
O Gar nicht	O Ein wenig	O Mäßig	O Ziemlich	O Sehr
**10b.** Während der letzten 7 Tage: Wie sehr hat Sie VERSCHWOMMENES SEHEN in Ihren täglichen Aktivitäten GESTÖRT?				
O Gar nicht	O Ein wenig	O Mäßig	O Ziemlich	O Sehr

**11a.** Während der letzten 7 Tage: Wie STARK waren Ihre PROBLEME SICH ZU KONZENTRIEREN im SCHLIMMSTEN FALL?				
O Gar nicht	O Ein wenig	O Mäßig	O Ziemlich	O Sehr
**11b.** Während der letzten 7 Tage: Wie sehr haben Ihre PROBLEME SICH ZU KONZENTRIEREN Sie in Ihren täglichen Aktivitäten GESTÖRT?				
O Gar nicht	O Ein wenig	O Mäßig	O Ziemlich	O Sehr

The PRO-CTCAE® items and information herein were developed by the Division of Cancer Control and Population Sciences in the NATIONAL CANCER INSTITUTE at the NATIONAL INSTITUTES OF HEALTH, in Bethesda, Maryland, U.S.A. Use of the PRO- CTCAE® is subject to NCI's Terms of Use.

**12a.** Während der letzten 7 Tage: Wie STARK waren Ihre PROBLEME MIT DEM GEDÄCHTNIS (VERGESSLICH-KEIT) im SCHLIMMSTEN FALL?				
O Gar nicht	O Ein wenig	O Mäßig	O Ziemlich	O Sehr
**12b.** Während der letzten 7 Tage: Wie sehr haben Sie Ihre PROBLEME MIT DEM GEDÄCHTNIS (VERGESSLICH-KEIT) in Ihren täglichen Aktivitäten GESTÖRT?				
O Gar nicht	O Ein wenig	O Mäßig	O Ziemlich	O Sehr

**13a.** Während der letzten 7 Tage: Wie HÄUFIG hatten Sie SCHMERZEN?				
O Nie	O Selten	O Gelegentlich	O Häufig	O Fast immer
**13b.** Während der letzten 7 Tage: Wie STARK waren Ihre SCHMERZEN im SCHLIMMSTEN FALL?				
O Gar nicht	O Ein wenig	O Mäßig	O Ziemlich	O Sehr
**13c.** Während der letzten 7 Tage: Wie sehr haben SCHMERZEN Sie in Ihren täglichen Aktivitäten GESTÖRT?				
O Gar nicht	O Ein wenig	O Mäßig	O Ziemlich	O Sehr

**14a.** Während der letzten 7 Tage: Wie STARK waren Ihre PROBLEME BEIM SCHLAFEN (WIE Z. B. SCHWIERIG-KEITEN BEIM EINSCHLAFEN, DURCHSCHLAFEN ODER ZU FRÜHES AUFWACHEN) im SCHLIMMSTEN FALL?				
O Gar nicht	O Ein wenig	O Mäßig	O Ziemlich	O Sehr
**14b.** Während der letzten 7 Tage: Wie sehr haben PROBLEME BEIM SCHLAFEN (WIE Z. B. SCHWIERIGKEITEN BEIM EINSCHLAFEN, DURCHSCHLAFEN ODER ZU FRÜHES AUFWACHEN) Sie in Ihren täglichen Aktivitäten GESTÖRT?				
O Gar nicht	O Ein wenig	O Mäßig	O Ziemlich	O Sehr

**15a.** Während der letzten 7 Tage: Wie STARK waren Ihre MÜDIGKEIT, ERSCHÖPFUNG ODER FEHLENDE ENER-GIE im SCHLIMMSTEN FALL?				
O Gar nicht	O Ein wenig	O Mäßig	O Ziemlich	O Sehr
**15b.** Während der letzten 7 Tage: Wie sehr haben MÜDIGKEIT, ERSCHÖPFUNG ODER FEHLENDE ENERGIE Sie in Ihren täglichen Aktivitäten GESTÖRT?				
O Gar nicht	O Ein wenig	O Mäßig	O Ziemlich	O Sehr

**16a.** Während der letzten 7 Tage: Wie HÄUFIG hatten Sie ANGST?				
O Nie	O Selten	O Gelegentlich	O Häufig	O Fast immer
**16b.** Während der letzten 7 Tage: Wie STARK war Ihre ANGST im SCHLIMMSTEN FALL?				
O Gar nicht	O Ein wenig	O Mäßig	O Ziemlich	O Sehr
**16c.** Während der letzten 7 Tage: Wie sehr hat ANGST Sie in Ihren täglichen Aktivitäten GESTÖRT?				
O Gar nicht	O Ein wenig	O Mäßig	O Ziemlich	O Sehr

The PRO-CTCAE® items and information herein were developed by the Division of Cancer Control and Population Sciences in the NATIONAL CANCER INSTITUTE at the NATIONAL INSTITUTES OF HEALTH, in Bethesda, Maryland, U.S.A. Use of the PRO- CTCAE® is subject to NCI's Terms of Use.

**17a.** Während der letzten 7 Tage: Wie HÄUFIG hatten Sie das GEFÜHL, DASS SIE NICHTS AUFMUNTERN KONNTE?				
O Nie	O Selten	O Gelegentlich	O Häufig	O Fast immer
**17b.** Während der letzten 7 Tage: Wie STARK hatten Sie das GEFÜHL, DASS SIE NICHTS AUFMUNTERN KONNTE, im SCHLIMMSTEN FALL?				
O Gar nicht	O Ein wenig	O Mäßig	O Ziemlich	O Sehr
**17c.** Während der letzten 7 Tage: Wie sehr hat Sie das GEFÜHL, DASS SIE NICHTS AUFMUNTERN KONNTE in Ihren täglichen Aktivitäten GESTÖRT?				
O Gar nicht	O Ein wenig	O Mäßig	O Ziemlich	O Sehr

**18a.** Während der letzten 7 Tage: Wie HÄUFIG waren Sie TRAURIG?				
O Nie	O Selten	O Gelegentlich	O Häufig	O Fast immer
**18b.** Während der letzten 7 Tage: Wie STARK war Ihre TRAURIGKEIT im SCHLIMMSTEN FALL?				
O Gar nicht	O Ein wenig	O Mäßig	O Ziemlich	O Sehr
**18c.** Während der letzten 7 Tage: Wie sehr hat Ihre TRAURIGKEIT Sie in Ihren täglichen Aktivitäten GESTÖRT?				
O Gar nicht	O Ein wenig	O Mäßig	O Ziemlich	O Sehr

**19a.** Während der letzten 7 Tage: Wie HÄUFIG hatten Sie SCHÜTTELFROST**?**				
O Nie	O Selten	O Gelegentlich	O Häufig	O Fast immer
**19b.** Während der letzten 7 Tage: Wie STARK war Ihr SCHÜTTELFROST im SCHLIMMSTEN FALL?				
O Gar nicht	O Ein wenig	O Mäßig	O Ziemlich	O Sehr

The PRO-CTCAE® items and information herein were developed by the Division of Cancer Control and Population Sciences in the NATIONAL CANCER INSTITUTE at the NATIONAL INSTITUTES OF HEALTH, in Bethesda, Maryland, U.S.A. Use of the PRO- CTCAE® is subject to NCI's Terms of Use.

WEITERE SYMPTOME	
Haben Sie weitere Symptome, die Sie benennen möchten?	
O Ja	O Nein
**Bitte nennen Sie alle weiteren Symptome:**	
1.	Während der letzten 7 Tage: Wie STARK hatten Sie das Symptom im SCHLIMMSTEN FALL?<br>O Gar nicht O Ein wenig O Mäßig O Ziemlich O Sehr
2.	Während der letzten 7 Tage: Wie STARK hatten Sie das Symptom im SCHLIMMSTEN FALL?<br>O Gar nicht O Ein wenig O Mäßig O Ziemlich O Sehr
3.	Während der letzten 7 Tage: Wie STARK hatten Sie das Symptom im SCHLIMMSTEN FALL?<br>O Gar nicht O Ein wenig O Mäßig O Ziemlich O Sehr
4.	Während der letzten 7 Tage: Wie STARK hatten Sie das Symptom im SCHLIMMSTEN FALL?<br>O Gar nicht O Ein wenig O Mäßig O Ziemlich O Sehr
5.	Während der letzten 7 Tage: Wie STARK hatten Sie das Symptom im SCHLIMMSTEN FALL?<br>O Gar nicht O Ein wenig O Mäßig O Ziemlich O Sehr

The PRO-CTCAE® items and information herein were developed by the Division of Cancer Control and Population Sciences in the NATIONAL CANCER INSTITUTE at the NATIONAL INSTITUTES OF HEALTH, in Bethesda, Maryland, U.S.A. Use of the PRO- CTCAE® is subject to NCI's Terms of Use.
