# Supplementary material for: Virtual reality support during systemic cancer therapy to improve anxiety/depressive symptoms and reduce toxicity in patients with gastrointestinal cancers—OncoVR
Source: ESMO Gastrointest Oncol. 2025 Feb 3;7:100135. doi: 10.1016/j.esmogo.2025.100135 (PMC12836744; doi:10.1016/j.esmogo.2025.100135)
Supplement: Supplementary Material [file mmc2.docx]

**Both Intensity and Duration of Blood Pressure Exposure are Associated with Mortality in Critically Ill Patients: A Retrospective Study**

Xiao-Yan Ding ^1^ ; Zhi-Zhong Chen ^2^ ; Han Chen ^1*^

1. The Fourth Department of Critical Care Medicine, Shengli Clinical Medical College of Fujian Medical University, Fuzhou University Affiliated Provincial Hospital, Fujian Provincial Hospital, Fujian Provincial Center for Critical Care Medicine, Fujian Provincial Key Laboratory of Critical Care Medicine, Fuzhou, Fujian, China.

2. General Product Center, Fujian Foxit Software Development Joint Stock Co. Ltd., Fuzhou, Fujian, China.

**Electronic Supplementary Material**

**SUPPLEMENTARY METHODS**

**Database**

All data in this study were obtained from the Medical Information Mart for Intensive Care (MIMIC)-IV database,^1^ an international freely accessible resource approved by the Institutional Review Board of the Massachusetts Institute of Technology. The MIMIC-IV database contains information for more than 50,000 patients admitted to the intensive care unit of Beth Israel Deaconess Medical Center between 2001 and 2019.

**Data extraction**

All data were extracted from the Medical Information Mart for Intensive Care (MIMIC-IV) database (version 2.0). The extracted data comprises the demographic information, co-morbidity diseases, and disease severity scores.^2^ Additionally, we extracted the MAP, systolic blood pressure (SBP), diastolic blood pressure (DBP), and the corresponding exposure duration for further calculating out-of-threshold events. Dr. Han Chen and Dr. Xiao-Yan Ding have been authorized to extract the data from the MIMIC database (certification number: HC 53297811, XYD 55860595). The study protocol was approved by the Institutional Review Board of Fujian Provincial Hospital.

**Inclusion and exclusion criteria**

The inclusion criteria were: 1) Age > 18 years; 2) blood pressure data available.

The exclusion criteria were: 1) blood pressure monitoring duration < 24 hours; 2) The second or subsequent admission to intensive care unit (ICU); 3) blood pressure data were not available. The following data were excluded from the analysis: 1) blood pressure records prior to the ICU admission; 2) blood pressure records after ICU discharge; 3) blood pressure records after 28 days of ICU stay.

**Missing data**

The missing values were calculated according to the formula MAP = 1/3SBP + 2/3DBP first if any two of the three variables were available.^3^ Subsequently, missing data were replaced by values of the same patients’ most adjacent available time points. The highest extreme MAP exceeding the 99^th^ percentile was replaced with a value slightly larger than the 99^th^ percentile (i.e., 130 mmHg), and the lowest extreme MAP less than the 1^st^ percentile was replaced with a value slightly smaller (i.e., 48 mmHg). Missing data for SBP/DBP and the outliers were handled by the same methods, and the corresponding range was 60 to 180 mmHg and 30 to 110 mmHg, respectively. The missing values of baseline data (e.g., height, weight, etc.) were replaced with the median or mean according to the distribution. The missing data accounted for less than 4% of the data analyzed.

**Visualization method**

All blood pressure values during the initial ICU admission were extracted, and the duration of each blood pressure value exceeding a predefined threshold was counted as an out-of-threshold event. The mean blood pressure exposure threshold was set between 48 and 130 mmHg and considered at 2-mmHg intervals. Different durations of exposure between 1 and 24 hours were considered at 1-hour intervals. Exposure event counts were computed for each patient across each duration (1-24 h) and the combination of MAP exposure (48-130 mmHg). The sum of out-of-threshold events at each MAP intensity-duration combination was recorded for both survivors and non-survivors. The event per survivor/non-survivor for each combination was then calculated by dividing the total count of events by the number of survivors/non-survivors. The OR was then calculated as the ratio between event per survivor and event per non-survivor. We calculated the OR deviation by subtracting the overall OR (ratio of the survivor to non-survivors) from the OR of each combination. The major modification of the current method to our previous studies^2,4^ was that by using the MAP of 65 mmHg as the dividing point, the out-of-threshold events and durations were recorded if MAP was lower than the thresholds for MAP ≤ 64 mmHg (Figure S1-B), while events were recorded if MAP was higher than the thresholds for MAP ≥ 66 mmHg (Figure S1-A), since either a too high or too low blood pressure is associated with poor outcomes. By doing so, we were able to consider both the risk of hypertension and hypotension exposures simultaneously. In addition, to better visualize these differences, we used a pure green color to highlight all combinations with OR deviation less than 0, which indicates beneficial outcomes.

**Statistical analysis**

Continuous variables were presented as mean and standard deviation or median and interquartile ranges (IQR) and were compared using a Student’s *t*-test or the Mann-Whitney *U* test, as appropriate. Categorical variables were presented as counts (percentages) and compared using the chi-square test or Fisher’s exact test. Based on the time-weighted average MAP calculated as previously described, ^2^ locally weighted scatter plot smoothing (LOWESS) and restricted cubic spline (RCS) regression model were performed to evaluate its impact on survival. Data manipulation and analysis were performed with STATA software (Version 18, StataCorp., TX, USA). The RCS was plotted by R studio (Version 4.3.3 Posit Software, PBC). The LOWESS plot and heatmap were plotted by Python (Version 3.12) with Matplotlib package (Version 3.8.0). All reported p-values were two-tailed, and a p-value less than 0.05 was considered statistically significant.

**SUPPLEMENTARY RESULTS**

A total of 41,602 patients (6,384,305 records) with blood pressure monitoring were included in the study (Figure S2), of whom the 28-day mortality rate was 12.8% (36,257 survivors and 5,345 non-survivors). As shown in Table S1, the non-survival group was older and more likely to be female. The non-survival group showed significantly higher sequential organ failure assessment (SOFA) score and acute physiology score III (APS-III) (7 [4, 11] vs. 4 [2, 6], p < 0.001, and 67 [48, 90] vs. 38 [29, 51], p < 0.001; respectively], compared with survivors. A higher incidence of rheumatic disease, liver disease, chronic pulmonary disease, myocardial infarction, cerebrovascular disease, and malignant cancer was observed in the non-survival group, while the incidence of hypertension was higher in survivors than in non-survivors (Table S1). The time-weighted average MAP was significantly lower in the non-survivor group than in the survivor group (75.1 [69.0, 82.7] vs. 78.5 [72.6, 85.9] mmHg, p < 0.001).

A curved quadrilateral region was highlighted in pure green when employing the alternative color scheme to emphasize areas OR deviation < 0 (Figure S3). Notably, we observed a dynamic relationship between MAP and mortality risk that depends on exposure duration. Even MAP values within the conventional normal range (66-80 mmHg) demonstrated a benefit-risk reversal phenomenon: shorter exposures were associated with mortality risk, while prolonged exposures showed survival benefit. This transition point varied with MAP levels - higher MAP values required shorter exposure durations to demonstrate benefit. Similar trends were observed in both the sepsis and hypertension subgroups and for both SBP and DBP (Figures S4 and S5). The selection of dividing points did not significantly alter the distribution of risk and benefit, although there were slight changes in the OR deviation value for each intensity-duration combination (Figures S8). This was because the change in dividing points altered the calculated overall OR value of the population. However, as the dividing point moved to the right, the risk of low blood pressure exposure was more prominently displayed, while the transition from mortality risk to survival benefit could not be fully depicted.

We defined the 'absolute safe zone' as the blood pressure range that consistently showed survival benefit (OR deviation < 0) regardless of exposure duration (i.e., all blocks were green in a column). For example, the “absolute safe zone” ranges from 82 to 110 mmHg in sepsis patients. In addition, these absolute safe zones varied among different populations. (Figures S3, S6 and S7).

Both the univariate analysis (LOWESS) and the multivariable analysis adjusted for age, gender, and SOFA score (RCS) revealed a “U”-shaped relationship with increasing time-weighted average MAP and 28-day mortality (Figure S9). Similarly, both systolic blood pressure and diastolic blood pressure exhibited this trend in relation to 28-day mortality (Figure S10).

**SUPPLEMENTARY TABLES**

**Table S1. Baseline characteristics of the total study population**

|  | | **All patients** | | | **Sepsis** | | | **Hypertension** | | |
| --- | --- | --- | --- | --- | --- | --- | --- | --- | --- | --- |
|  | **Survior (n = 36,257)** | | **Non-Survior  (n = 5,345)** | ***P*** | **Survior (n = 17,289)** | **Non-Survior (n = 3,821)** | ***P*** | **Survior (n = 20,449)** | **Non-Survior (n = 2,649)** | ***P*** |
| Age (years) | 66.2 [54.4, 77.0] | | 75.1 [62.5, 84.6] | < 0.001 | 66.9 [55.7, 77.5] | 73.8 [61.4, 83.5] | < 0.001 | 68.7 [59.3, 78.4] | 76.8 [65.8, 85.1] | < 0.001 |
| Weight (kg) | 80.4 [67.9, 95.7] | | 75.7 [62.6, 90] | < 0.001 | 81.6 [68.8, 97.4] | 77 [64.3, 92] | < 0.001 | 82.2 [69.3, 97.7] | 76 [62.4, 90] | < 0.001 |
| Hight (m) | 1.70 [1.69, 1.73] | | 1.70 [1.65, 1.70] | < 0.001 | 1.70 [1.68, 1.73] | 1.70 [1.65, 1.73] | < 0.001 | 1.70 [1.68, 1.73] | 1.70 [1.65, 1.70] | < 0.001 |
| BMI (kg/m^2^) | 27.8 [23.9, 32.7] | | 26.4 [22.4, 31.1] | < 0.001 | 28.2 [24.2, 33.1] | 26.9 [22.8, 31.9] | < 0.001 | 28.4 [24.4, 33.3] | 26.5 [22.6, 31.2] | < 0.001 |
| Male, n (%) | 20,726 (57.2%) | | 2,832 (53%) | < 0.001 | 10,175 (58.9%) | 2,100 (55.0%) | < 0.001 | 11,661 (57%) | 1,304 (49.2%) | < 0.001 |
| SOFA score | 4 [2, 6] | | 7 [4, 11] | < 0.001 | 5 [4, 8] | 8 [6, 12] | < 0.001 | 4 [2, 6] | 7 [4, 10] | < 0.001 |
| APS-Ⅲ score | 38 [29, 51] | | 67 [48, 90] | < 0.001 | 46 [34, 62] | 73 [55, 95] | < 0.001 | 38 [29, 50] | 64 [46, 85] | < 0.001 |
| **Co-morbidities** |  | |  |  |  |  |  |  |  |  |
| Rheumatic disease, n (%) | 1,160 (3.2%) | | 203 (3.8%) | 0.022 | 604 (3.5%) | 153 (4%) | 0.116 | 709 (3.5%) | 102 (3.9%) | 0.313 |
| Severe liver disease, n (%) | 1,342 (3.7%) | | 583 (10.9%) | < 0.001 | 999 (5.8%) | 480 (12.6%) | < 0.001 | 649 (3.2%) | 211 (8.0%) | < 0.001 |
| Chronic pulmonary disease, n (%) | 8,482 (23.4%) | | 1,443 (27.0%) | < 0.001 | 4,388 (25.4%) | 1,081 (28.3%) | 0.001 | 4,994 (24.4%) | 710 (26.8%) | 0.008 |
| Myocardial infarct, n (%) | 5,985 (16.5%) | | 1,072 (20.1%) | < 0.001 | 2,871 (16.6%) | 785 (20.5%) | < 0.001 | 3,678 (18%) | 501 (18.9%) | 0.244 |
| Hypertension, (%) | 20,449 (56.4%) | | 2,649 (49.6%) | < 0.001 | 9,764 (56.5%) | 1,795 (47.0%) | < 0.001 | / | / | / |
| Congestive heart failure, n (%) | 8,714 (24%) | | 1,793 (33.6%) | < 0.001 | 4,727 (27.3%) | 1,336 (35.0%) | < 0.001 | 4,458 (21.8%) | 711 (26.8%) | < 0.001 |
| Cerebrovascular disease, n (%) | 5,818 (16.1%) | | 1,309 (24.5%) | < 0.001 | 2,301 (13.3%) | 795 (20.8%) | < 0.001 | 3,741 (18.3%) | 800 (30.2%) | < 0.001 |
| Mild liver disease, n (%) | 3,399 (9.4%) | | 1,013 (19.0%) | < 0.001 | 2,176 (12.6%) | 841 (22%) | < 0.001 | 1,745 (8.5%) | 420 (15.9%) | < 0.001 |
| Diabetes mellitus with complications, n (%) | 3,031 (8.4%) | | 447 (8.4%) | 0.994 | 1554 (9%) | 350 (9.2%) | 0.738 | 1,472 (7.2%) | 159 (26.2%) | 0.024 |
| Diabetes mellitus without complications, n (%) | 8,145 (22.5%) | | 1,233 (23.1%) | 0.324 | 4,143 (24%) | 909 (23.8%) | 0.82 | 5,600 (27.4%) | 694 (6.0%) | 0.197 |
| Malignant cancer, n (%) | 4,087 (11.3%) | | 1,210 (22.6%) | < 0.001 | 1,975 (11.4%) | 810 (21.2%) | < 0.001 | 2,343 (11.5%) | 630 (23.8%) | < 0.001 |
| time-weighted average of SBP (mmHg) | 119.2 [110.5, 130.2] | | 114.4 [104.6, 127.7] | < 0.001 | 118.1 [109.6, 128.3] | 112.4 [103.6, 124.0] | < 0.001 | 121 [112.1, 132.4] | 117.1 [106.4, 131] | < 0.001 |
| time-weighted average of DBP (mmHg) | 63.2 [57.1, 70.6] | | 59.9 [53.5, 67.0] | < 0.001 | 61.6 [56.1, 68.1] | 59 [62.9, 65.9] | < 0.001 | 62.9 [56.7, 70.6] | 60.4 [54.1, 67.5] | < 0.001 |
| time-weighted average of MAP (mmHg) | 78.5 [72.6, 85.9] | | 75.1 [69.0, 82.7] | < 0.001 | 77.2 [71.9, 83.7] | 74.2 [68.4, 81.2] | < 0.001 | 78.8 [72.9, 86.6] | 76.2 [69.8, 83.6] | < 0.001 |

APS-III score: acute physiology score III; BMI: body mass index; DBP: diastolic blood pressure; MAP: mean artery blood pressure; SBP: systolic blood pressure; SOFA: sequential organ failure assessment.

**SUPPLEMENTARY FIGURES**

**Figure S1. The visualization methods**


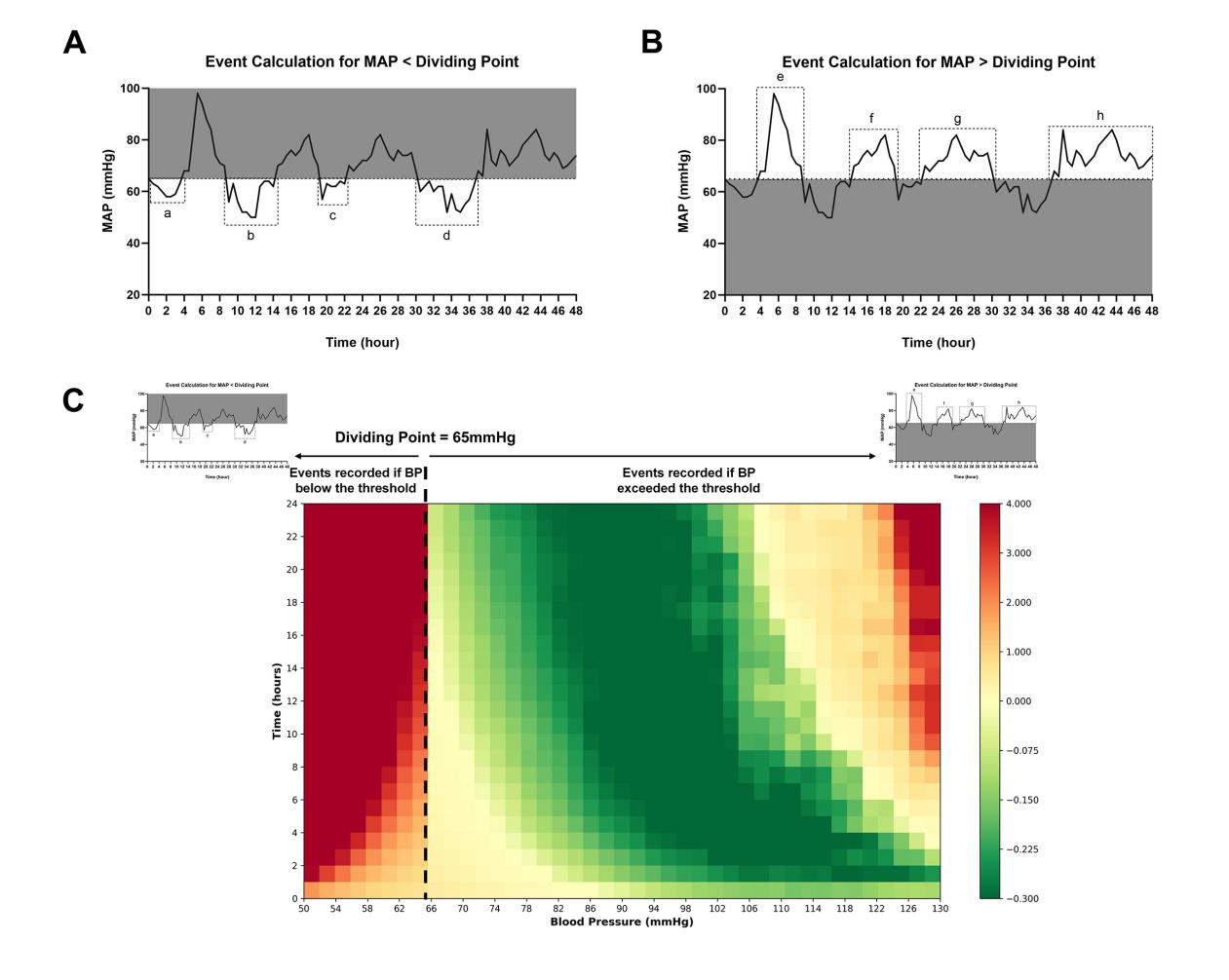


**Panel A:** Diagram for the calculation of the event count below the mean arterial pressure (MAP) dividing point. The line represents data from a patient. For instance, with 65 mmHg as the selected threshold, there could be 4 exposures with varying durations (*a-d*). The threshold selection was repeated in this patient in decrements of 2 mmHg from 64 to 48 mmHg and thus generated various event counts according to the various MAP thresholds. **Panel B:** For MAP greater than 65 mmHg, the frequency of out-of-threshold events and the corresponding duration were also recorded, where a MAP greater than the selected threshold was considered an event (*e-h*). The process was also repeated at varying thresholds (66-130 mmHg). Kindly note that the procedure was repeated in all patients, and thus provided the event counts at each MAP intensity-duration combination. **Panel C:** For each MAP intensity-duration combination, the event per non-survivor was divided by the event per survivor, which was the odds ratio (OR). The OR deviation was then calculated by subtracting the overall OR from the ORs of each combination, and a heatmap was utilized to visually represent the association between various blood pressure intensity-duration combinations and 28-day mortality combinations. The exposure duration ranged from 1 to 24 hours at 1-hour intervals displayed on the y-axis, while MAP ranged from 48 to 130 mmHg at 2-mmHg intervals displayed on the x-axis. Each intersection represents the OR deviation value for that intensity-duration combination, indicated by different colors. The black dashed line at 65 mmHg represents a key methodological distinction in exposure event calculation. For MAP values below this line (<65mmHg), events were counted when blood pressure falls below the threshold value, while for MAP values above this line (>65mmHg), events were counted when blood pressure exceeds the threshold value.

**Figure S2. The flow chart of patient selection**


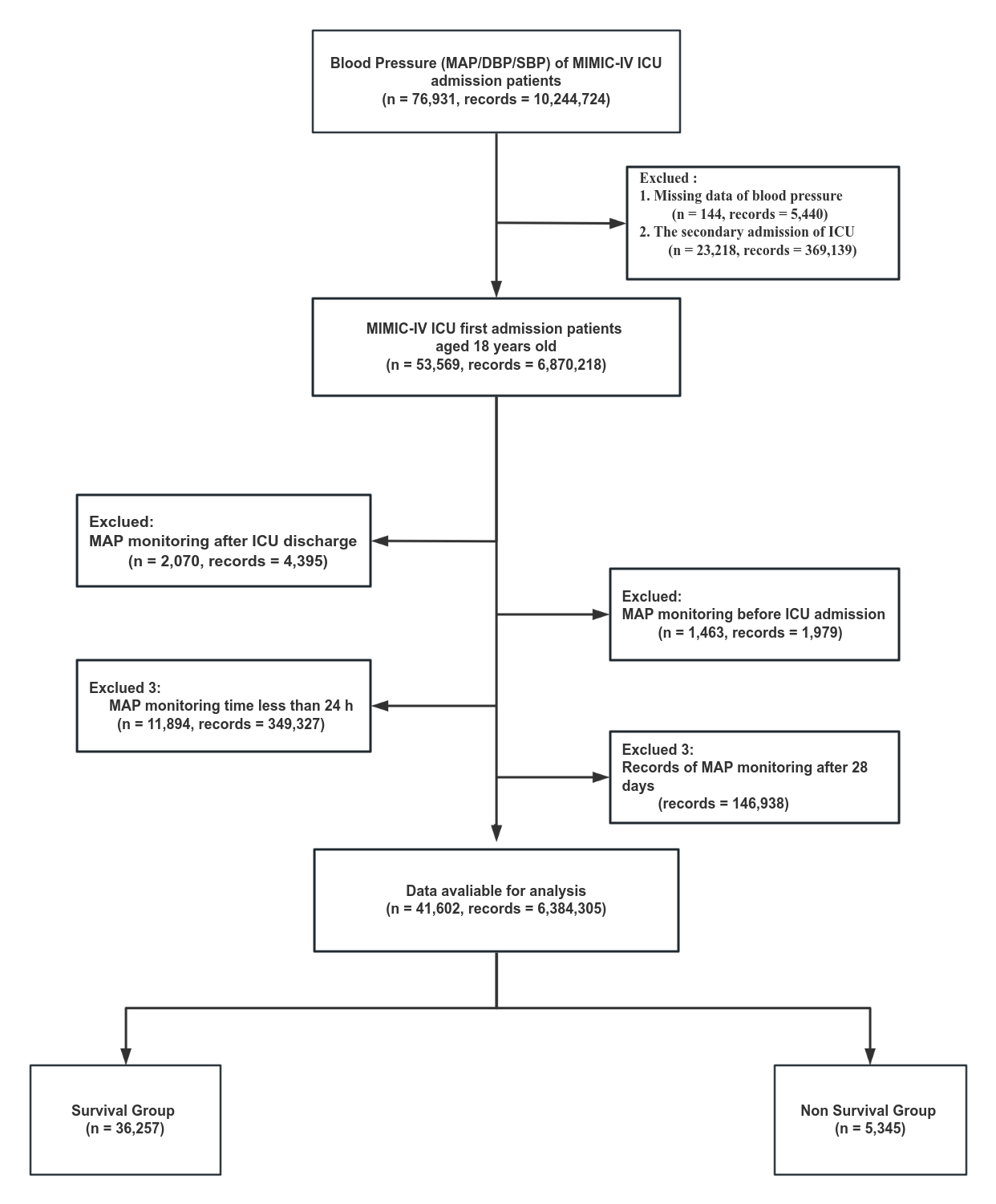


MIMIC-IV: Medical Information Mart for Intensive Care; ICU: intensive care unit; MAP: mean arterial pressure; SBP: systolic blood pressure; DBP: diastolic blood pressure.

**Figure S3. Variety "absolute safe zone" of mean arterial pressure among different populations**


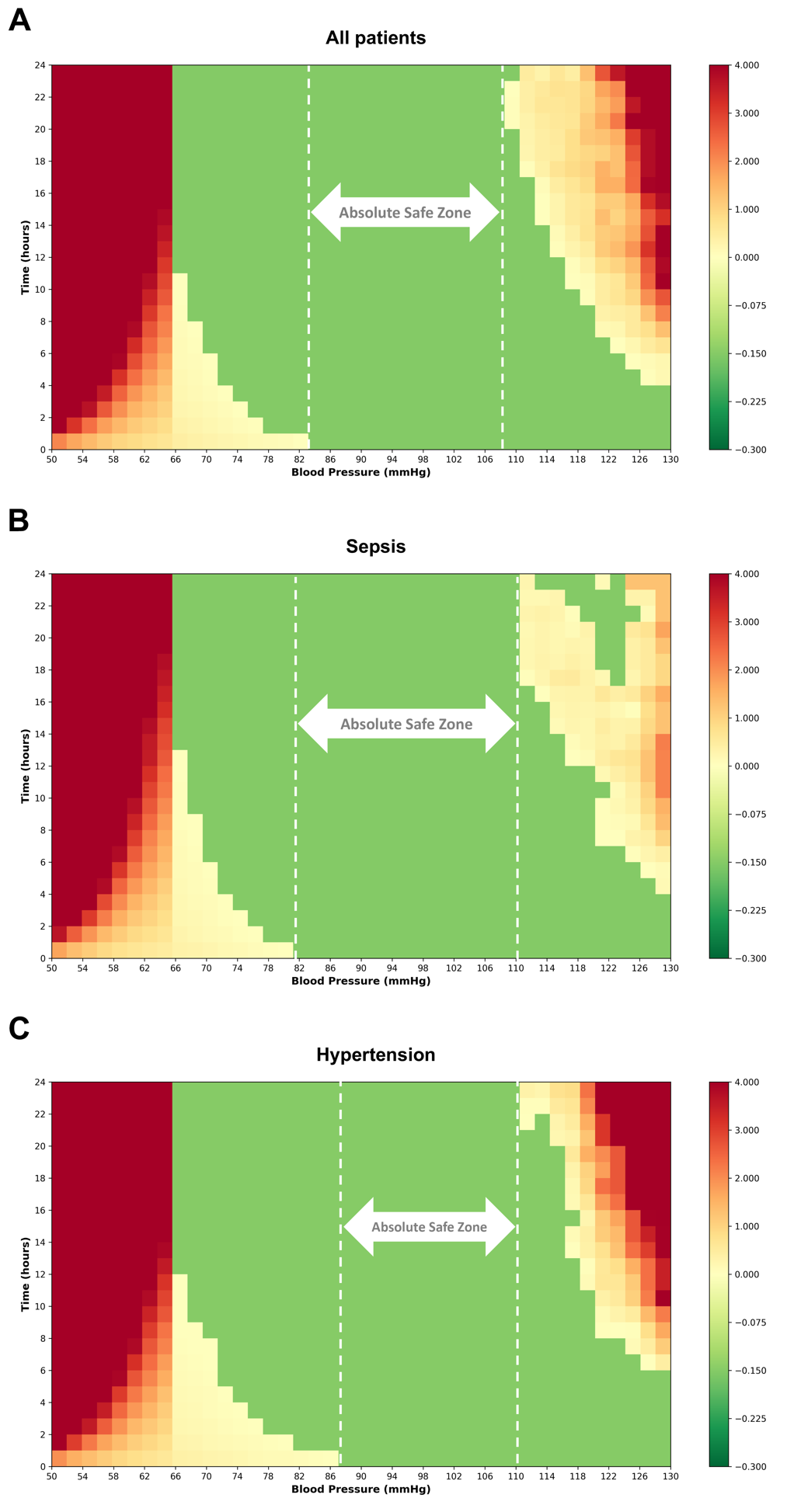


All blocks with an odds ratio deviation < 0 were filled with the same green color to highlight the combinations associated with survival benefit. Additionally, we defined and marked the “absolute safe zone” - blood pressure ranges that consistently showed survival benefit regardless of exposure duration. Notably, these absolute safe zones varied among different populations. *Penal A*: overall population, *Penal B*: sepsis, *Penal C*: hypertension.

**Figure S4 Heatmap illustrating the odds ratio deviation of the systolic blood pressure exposure**


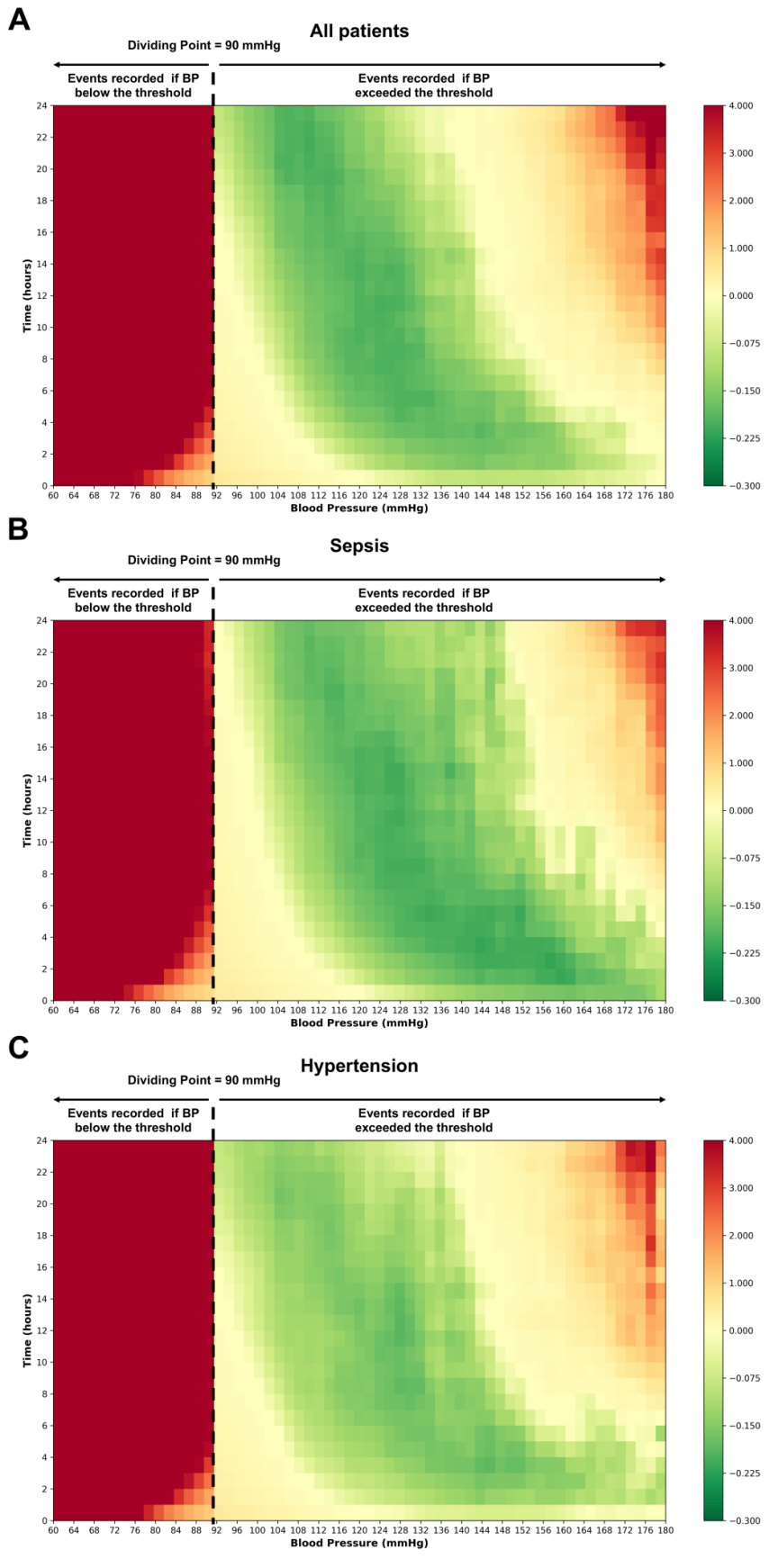


***Penal A*:** The dividing point of 90 mmHg was used to visualize the risk for MAP ≤ 90 mmHg and > 90 mmHg, respectively. The green area indicates OR deviation for favorable effects, and the red area indicates unfavorable effects. The trend was also observed in sepsis (*Penal B*) and hypertension (*Penal C*) populations.

**Figure S5 Heatmap illustrating the odds ratio deviation of the diastolic blood pressure exposure**


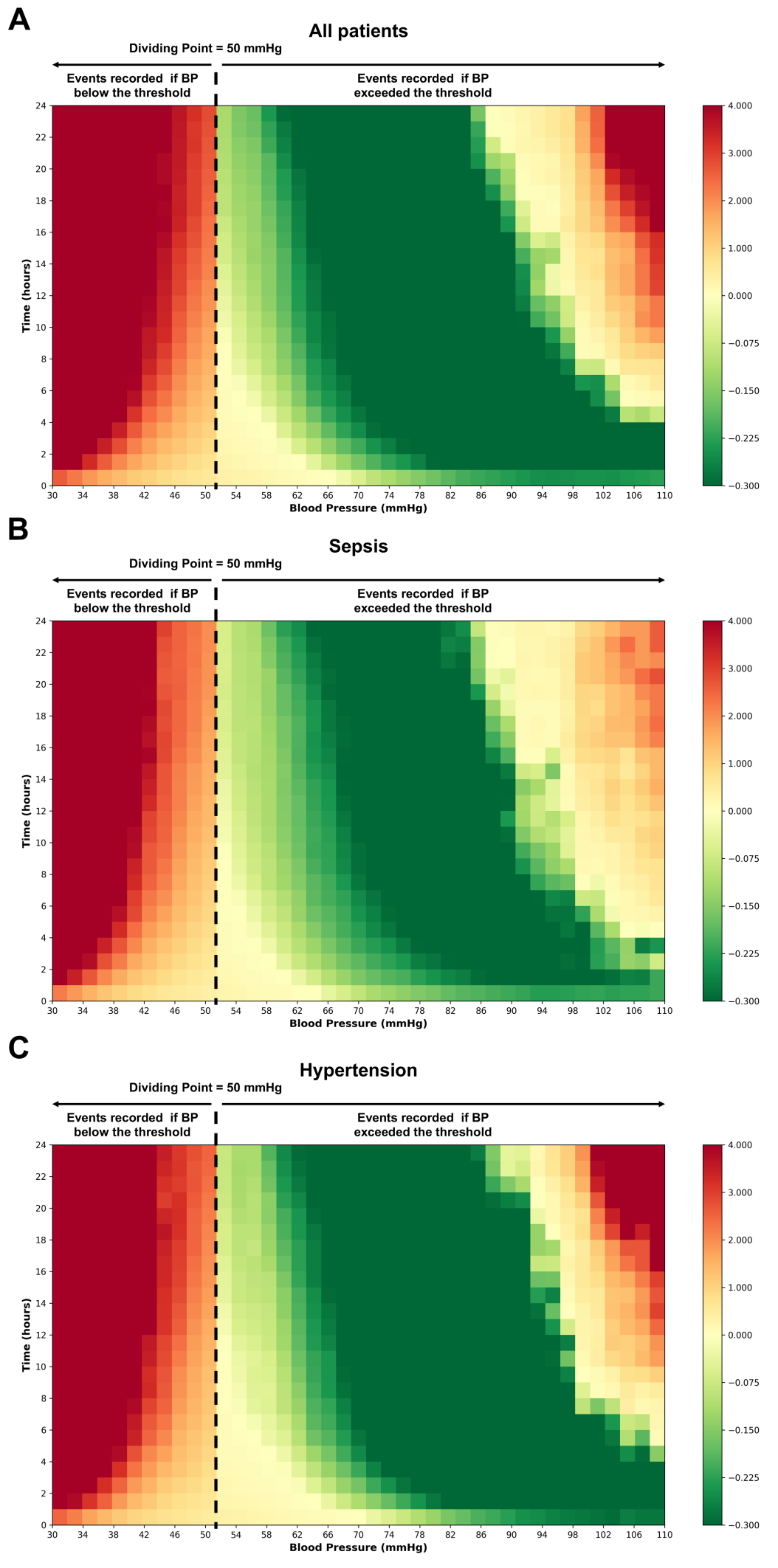


**Penal A:** The dividing point of 50 mmHg was used to visualize the risk ratios for MAP ≤ 50 mmHg and > 50 mmHg, respectively. The trend was also observed in sepsis (*Penal B*) and hypertension (*Penal C*) populations.

**Figure S6 Variety "absolute safe zone" of systolic blood pressure among different populations**


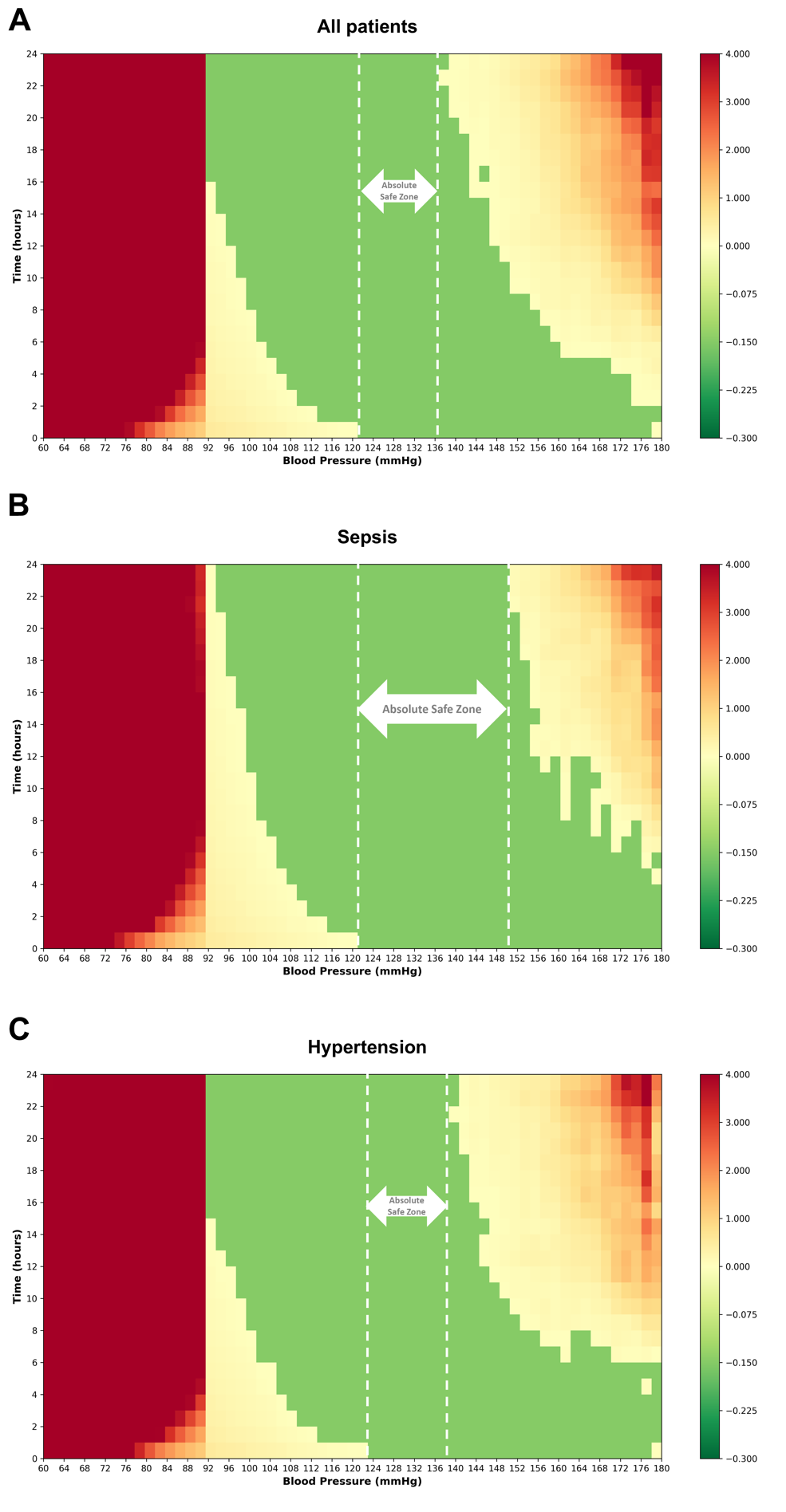


Similar to mean arterial pressure, absolute safe zones can also be observed in systolic blood pressure, although with more pronounced differences - sepsis patients demonstrated a wider range. *Penal A*: overall population, *Penal B*: sepsis, *Penal C*: hypertension.

**Figure S7 Variety "absolute safe zone" of diastolic blood pressure among different populations.**


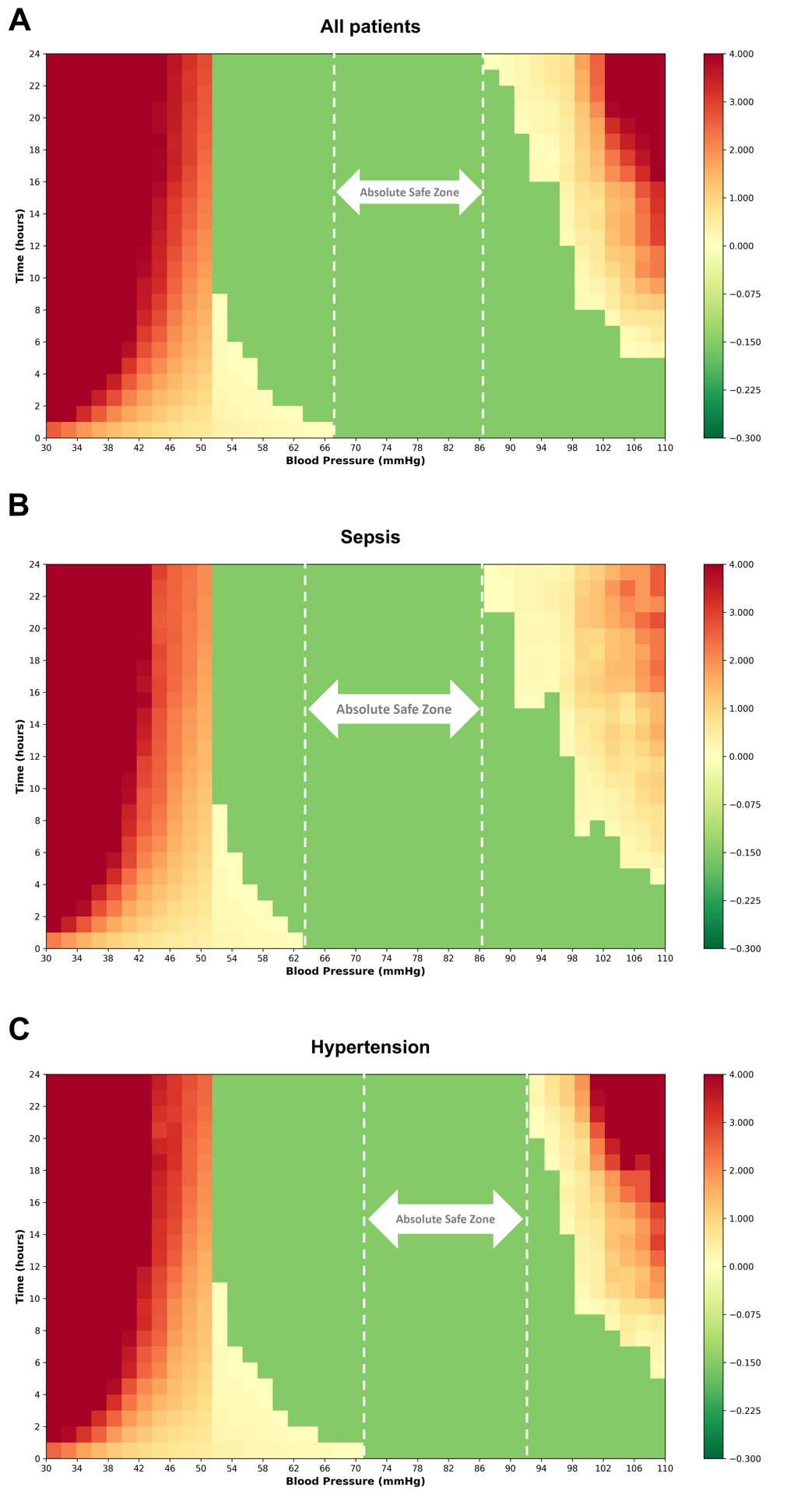


Similar to mean arterial pressure and systolic blood pressure, absolute safe zones can also be observed in diastolic blood pressure. *Penal A*: overall population, *Penal B*: sepsis, *Penal C*: hypertension.

**Figure S8 Comparison of different selections of dividing points for diastolic blood pressure to the distribution of risk/benefit**


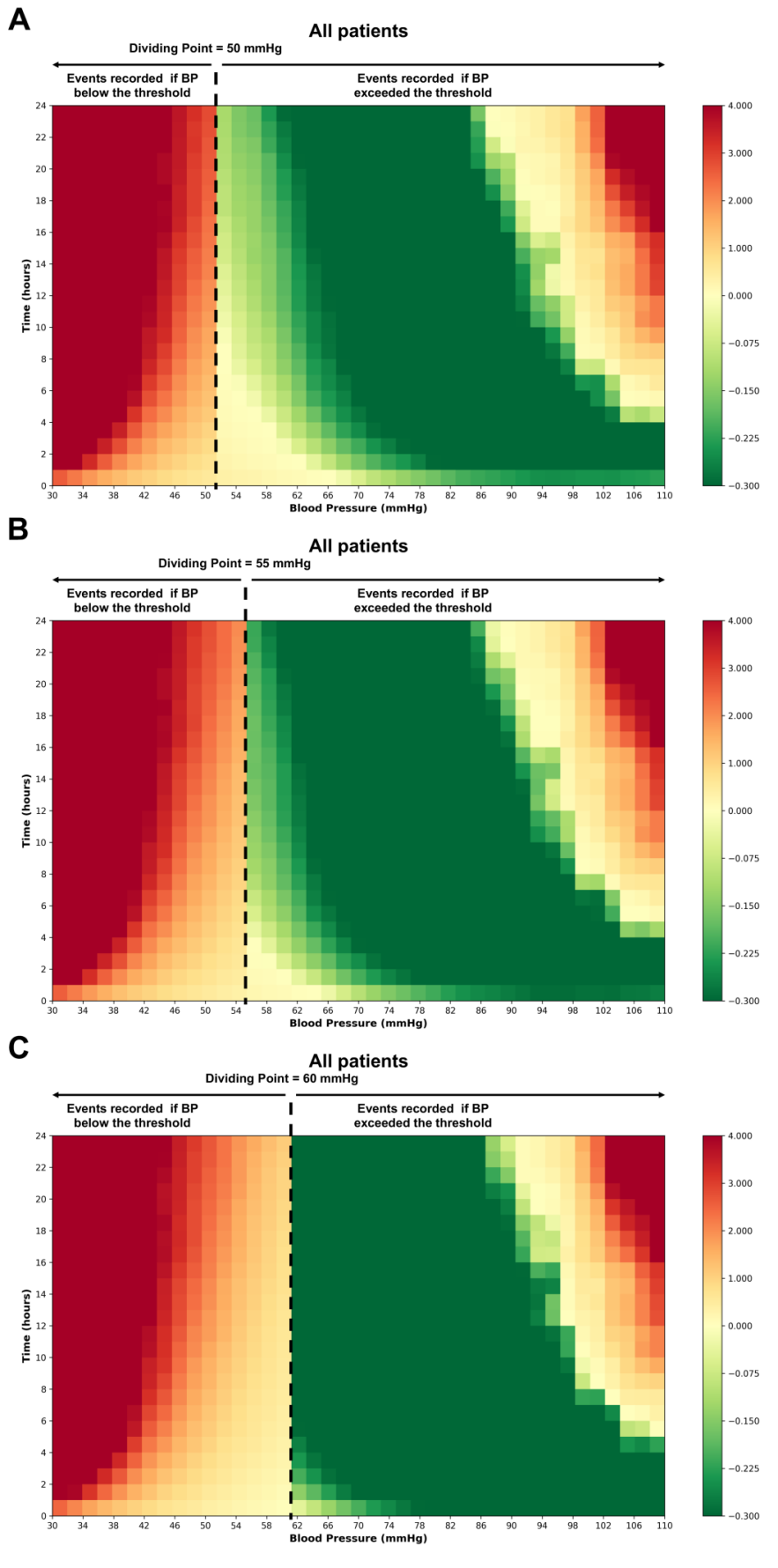


The visualization method was applied to diastolic blood pressure, with dividing points of 50 mmHg, 55 mmHg, and 60 mmHg, respectively. The left border of the “green zone” was masked as the dividing point setting was raised. However, the selection of the dividing point did not alter the distribution of the green area.

**Figure S9. Univariate and multivariate analyses of the relationship between time-weighted average mean blood pressure values and 28-day mortality**

**
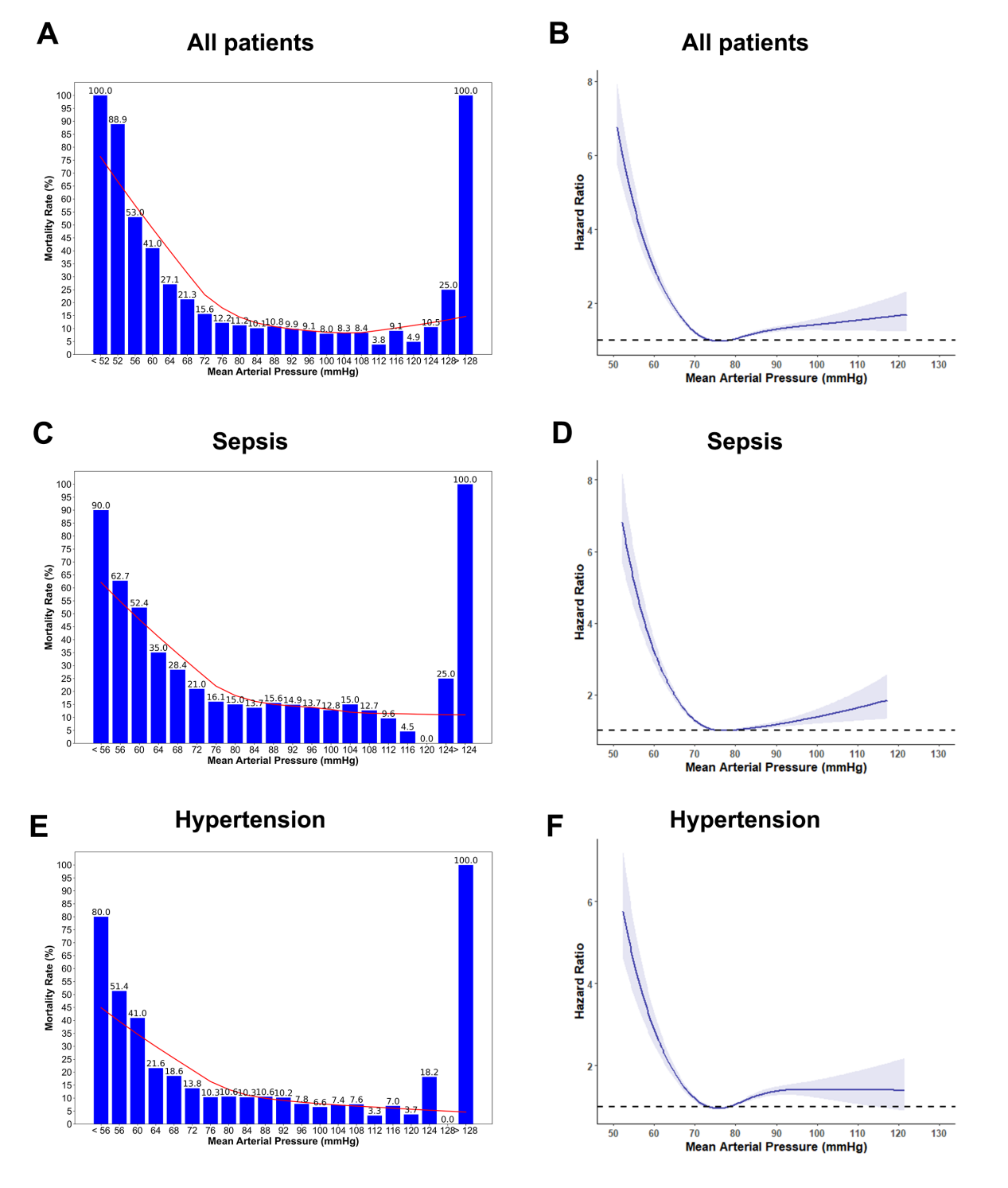
**

Locally weighted scatter plot smoothing (LOWESS) was used as the univariate analysis, and the restricted cubic spline (RCS) regression model was used as the multivariate analysis. The association between time-weighted average mean blood pressure and 28-day mortality was explored. Both the univariate analysis (LOWESS, panels A, C, and E) and the multivariable analysis adjusted for age, gender, and sequential organ failure assessment score (RCS, panels B, D, and F) revealed a near “U”-shaped relationship with increasing time-weighted average MAP and 28-day mortality. The trend could be observed in the overall, sepsis and hypertension populations.

**Figure S10. Univariate and multivariate analysis of the crude relationship between blood pressure and 28-day mortality in the overall population**


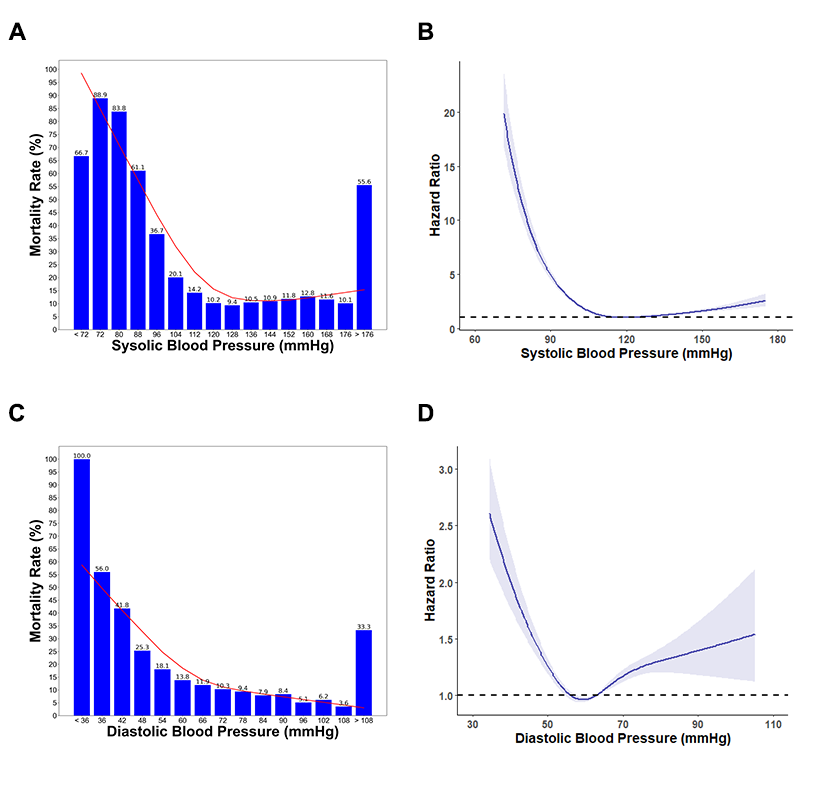


Both time-weighted average systolic blood pressure and time-weighted average diastolic blood pressure showed a near U-shaped pattern in relation to 28-day mortality in the overall population.

**INTERPRETATION**

The main founding of our study were: 1) The relationship between time-weighted average MAP and 28-day mortality rate exhibited a U-shaped pattern, indicating that both excessively high and low blood pressure levels were associated with an increased risk of mortality; 2) such association was also confirmed when both the intensity and duration of blood pressure exposure were taken into consider using a heatmap for visualization; 3) however, for blood pressure levels near the lower or upper limits of the "normal" range, the benefits or risks depended on the duration of exposure (e.g., the "benefit-risk reversal phenomenon"); 4) the safe blood pressure intensity-duration combination roughly distributed in an approximate curved quadrilateral region, which may vary between different populations.

The target of optimal blood pressure is in constant controversy, and numerous studies aimed at determining optimal blood pressure levels and its derived parameters (e.g., cerebral perfusion pressure, myocardial perfusion pressure, renal perfusion pressure, etc.) have been conducted in various diseases, such as post-operative management of thrombectomy, resuscitation for septic shock, or maintaining suitable blood pressure after myocardial infarction.^5-8^ However, the majority of these studies have primarily focused on values, neglecting the potential impact of the duration of blood pressure exposure. In the current study, a conventional approach of calculating time-weighted averages in order to illustrate a raw correlation between the duration of blood pressure exposure and its subsequent impact on patient outcomes was applied. Our results revealed a U-shaped distribution of mortality risk, with LOWESS for univariate analysis and RCS for multivariate analysis. These findings confirmed previous works, emphasizing that both excessively high and low blood pressure are associated with poor outcomes.

However, the aforementioned findings were unsurprising and yet insufficient. The calculation of time-weighted averages failed to account for crucial factors influencing prognosis, such as fluctuations in blood pressure, the extremities of blood pressure levels, and, most importantly, the duration of each fluctuation. The human body employs compensatory mechanisms to uphold homeostasis, and fluctuations over a specific timeframe may not necessarily compromise organ function. However, prolonged fluctuations may lead to reversible or even irreversible effects, thereby impacting prognosis. Therefore, considering the duration of exposure is crucial. In light of this, we adopted and modified an analysis approach that previously successfully revealed the relationship between mortality and intracranial pressure^4^ or dynamic mechanical power.^2^

We identified mortality risks within the conventional “normal” blood pressure range. Lower MAP values (e.g., 66 mmHg), despite exceeding typical septic shock resuscitation targets, were associated with mortality when maintained for ≤12 hours. This suggests that achieving target blood pressure alone is insufficient without adequate duration maintenance, possibly reflecting impaired homeostasis due to disease severity, organ dysfunction, or inadequate treatment. Similar risks were observed at the upper limit of normal blood pressure range. Our visualization method effectively demonstrated this intensity-duration relationship with mortality.

Another interesting finding was the “absolute safe zone”. This finding yields two implications: first, for individuals with stable hemodynamics or those in good health, blood pressure can naturally maintain a normal range to uphold the body's physiological functions, the duration of exposure is thus not correlated with adverse outcomes. Second, this may partially explain the discrepancy in previous studies. Vincent *et al.* observed no correlation between hypotension and mortality with a target of 80 mmHg in sepsis patients.^9^ Marco *et al.* determined that a MAP of 81.5 mmHg or lower was the optimal threshold for predicting clinical deterioration within 48 hours.^10^ Chen *et al.* identified the optimal MAP range as 80-90 mmHg for moderate and high-risk pulmonary embolism patients.^11^ Collectively, these studies imply that MAP targets higher than 80 mmHg were no longer linked to improved outcomes since such targets were located in the “absolute safe zone” identified by the current study. Conversely, for MAP targets below 80 mmHg, different studies have yielded varied conclusions. Maheshwari *et al.* found no difference in mortality rates between lower blood pressure targets of 65-70 mmHg.^12^ The 65 trial also indicated that permissive hypotension (MAP target 60-65 mmHg) did not lead to a significant difference in mortality among patients aged 65 years or older.^13^ These results contrast with the previously mentioned studies, which suggest that lower MAP targets were associated with increased mortality risk.^10-14^ The discrepancies in previous research can be partially explained by our findings, which revealed the benefit/risk reversal phenomenon within such MAP range, with the duration of MAP exposure identified as the pivotal factor for this reversal. The inadequate consideration of duration in past studies contributed to such discrepancies. It is important to note that our findings do not advocate for the establishment of higher blood pressure targets to achieve an "absolutely safe zone." Instead, we emphasize the significance of exposure duration, a metric that has not been comprehensively explored. Our findings provide new insights for future research in this field.

Some previous studies also considered the duration of blood pressure exposure by employing a variety of different methods. One way is to calculate the time-weighted average.^15-17^ As discussed previously, this method smooths over the fluctuations and obscures the true episode of low or high blood pressure, while the fluctuations are often more relevant to adverse outcomes.^8^ Another approach was to calculate the cumulative exposure time below given MAP thresholds, which has been utilized in sepsis and non-cardiac surgery.^12,14,18^ However, in these studies, the blood pressure threshold was set at relatively wide intervals (55mmHg, 65mmHg, 75mmHg, and 85mmHg) and considered the limited exposure duration of 120 minutes. An additional similar approach involves calculating the maximum duration of exposures below a threshold rather than cumulative duration.^9^ In addition to the inherent limitation of the selected thresholds (considering MAP < 80, < 75, < 65, < 60, and < 55 mmHg in this study), this method only considered the longest exposure within a 24-hour period, thereby overlooking the potential impacts of shorter exposures. Our study exposed the duration at 1-hour intervals, which provided higher temporal precision. Besides, we explored a broader MAP range from 48 to 130 mmHg at a 2-mmHg interval. Moreover, our calculation exhaustively traversed all combinations of intensity and duration, utilizing all available blood pressure data for each combination and maximizing the utilization of the dataset resources. This approach allowed us to create a heatmap that not only illustrates the relationship between intensity-duration combinations and mortality but also provides insights into their distribution patterns. In summary, we have introduced a novel method that can be used to explore the dose-effect relationship, enhancing our understanding of the complex interactions among blood pressure exposure intensity, duration, and outcomes.

There are several limitations in our study. First, this was a retrospective study based on the MIMIC-IV database, precluding the establishment of definitive causal relationships. While our approach of visualizing hypotension "dose" breaks through traditional research limitations by comprehensively considering exposure intensity and duration, the observed increase in mortality requires validation through large-scale prospective studies. Therefore, clinicians should exercise caution when interpreting these results and continue to rely on comprehensive clinical assessment rather than using these findings in isolation. Second, while we performed both univariate and multivariate analyses adjusting for basic confounders (age, gender, and SOFA score), our heatmap visualization remains primarily a univariate analysis tool that does not account for other important factors such as vasoactive medications, fluid resuscitation, and patient-specific comorbidities. Therefore, this visualization method should be viewed as a warning tool rather than a precise predictor of mortality risk. Future studies should incorporate more comprehensive multivariate models to better account for these confounding variables. Third, we included all blood pressure values without differentiating between invasive and non-invasive measurements or the site of blood pressure measurement (e.g., radial vs. femoral artery). This represents a significant limitation as these different measurement methods can yield markedly different results, particularly in critically ill patients. This methodological limitation could introduce measurement bias in our findings.

**SUPPLEMENTARY REFERENCES**

1. Johnson A BL, Pollard T, Horng S, Celi LA, Mark R. "MIMIC-IV" (ver‑sion 1.0). PhysioNet 2021. DOI: <https://doi.org/10.13026/6mm1-ek67>.

2. Chen H, Chen Z-Z, Gong S-R, Yu R-G. Visualizing the dynamic mechanical power and time burden of mechanical ventilation patients: an analysis of the MIMIC-IV database. Journal of Intensive Care 2023;11(1):58. DOI: 10.1186/s40560-023-00709-9.

3. Zuin M, Rigatelli G, Bongarzoni A, et al. Mean arterial pressure predicts 48 h clinical deterioration in intermediate-high risk patients with acute pulmonary embolism. Eur Heart J Acute Cardiovasc Care 2023;12(2):80-86. (In eng). DOI: 10.1093/ehjacc/zuac169.

4. Ding XY, Chen ZZ, Chen H. Visualizing ICP "Dose" of neurological critical care patients. Intensive Care Med 2024;50(5):781-783. (In eng). DOI: 10.1007/s00134-024-07424-5.

5. Wartenberg KE, Mayer SA. Determining the optimal target blood pressure after thrombectomy: High or low? Neurology 2017;89(6):528-529. (In eng). DOI: 10.1212/WNL.0000000000004188.

6. Maiwall R, Rao Pasupuleti SS, Hidam AK, et al. A randomised-controlled trial (TARGET-C) of high vs. low target mean arterial pressure in patients with cirrhosis and septic shock. J Hepatol 2023;79(2):349-361. (In eng). DOI: 10.1016/j.jhep.2023.04.006.

7. Menon V. Targeting Mean Arterial Pressure to Limit Myocardial Injury: Novel Finding or Wild Goose Chase? J Am Coll Cardiol 2020;76(7):825-827. (In eng). DOI: 10.1016/j.jacc.2020.06.054.

8. Stulberg EL, Harris BRE, Zheutlin AR, et al. Association of Blood Pressure Variability With Death and Discharge Destination Among Critically Ill Patients With and Without Stroke. Neurology 2023;101(11):e1145-e1157. (In eng). DOI: 10.1212/WNL.0000000000207599.

9. Vincent JL, Nielsen ND, Shapiro NI, et al. Mean arterial pressure and mortality in patients with distributive shock: a retrospective analysis of the MIMIC-III database. Ann Intensive Care 2018;8(1):107. (In eng). DOI: 10.1186/s13613-018-0448-9.

10. Zuin M, Rigatelli G, Bongarzoni A, et al. Mean arterial pressure predicts 48 h clinical deterioration in intermediate-high risk patients with acute pulmonary embolism. Eur Heart J Acute Cardiovasc Care 2023;12(2):80-86. (In eng). DOI: 10.1093/ehjacc/zuac169.

11. Chen J, Lin J, Wu D, Guo X, Li X, Shi S. Optimal Mean Arterial Pressure Within 24 Hours of Admission for Patients With Intermediate-Risk and High-Risk Pulmonary Embolism. Clin Appl Thromb Hemost 2020;26:1076029620933944. (In eng). DOI: 10.1177/1076029620933944.

12. Maheshwari K, Nathanson BH, Munson SH, et al. The relationship between ICU hypotension and in-hospital mortality and morbidity in septic patients. Intensive Care Med 2018;44(6):857-867. (In eng). DOI: 10.1007/s00134-018-5218-5.

13. de Havenon A, Petersen NH, Stulberg EL, Anadani M, Biffi A, Sheth KN. Interaction of Mean Arterial Pressure and Blood Pressure Variability in Critically Ill Brain Injured Patients. Stroke 2022;53(12):e512-e514. (In eng). DOI: 10.1161/STROKEAHA.122.041274.

14. Khanna AK, Kinoshita T, Natarajan A, et al. Association of systolic, diastolic, mean, and pulse pressure with morbidity and mortality in septic ICU patients: a nationwide observational study. Ann Intensive Care 2023;13(1):9. (In eng). DOI: 10.1186/s13613-023-01101-4.

15. Dünser MW, Takala J, Ulmer H, et al. Arterial blood pressure during early sepsis and outcome. Intensive Care Med 2009;35(7):1225-33. (In eng). DOI: 10.1007/s00134-009-1427-2.

16. Patidar KR, Peng JL, Pike F, et al. Associations Between Mean Arterial Pressure and Poor ICU Outcomes in Critically Ill Patients With Cirrhosis: Is 65 The Sweet Spot? Critical Care Medicine 2020;48(9):e753-e760. (In eng). DOI: 10.1097/CCM.0000000000004442.

17. Ameloot K, Jakkula P, Hästbacka J, et al. Optimum Blood Pressure in Patients With Shock After Acute Myocardial Infarction and Cardiac Arrest. Journal of the American College of Cardiology 2020;76(7):812-824. (In eng). DOI: 10.1016/j.jacc.2020.06.043.

18. Ahuja S, Mascha EJ, Yang D, et al. Associations of Intraoperative Radial Arterial Systolic, Diastolic, Mean, and Pulse Pressures with Myocardial and Acute Kidney Injury after Noncardiac Surgery: A Retrospective Cohort Analysis. Anesthesiology 2020;132(2):291-306. (In eng). DOI: 10.1097/aln.0000000000003048.
